# Supplementary material for: Application of Machine Learning Algorithms to Predict Uncontrolled Diabetes Using the All of Us Research Program Data
Source: Healthcare (Basel). 2023 Apr 15;11(8):1138. doi: 10.3390/healthcare11081138 (PMC10137945; doi:10.3390/healthcare11081138)
Supplement: Supplementary file 1 [file healthcare-11-01138-s001.zip › healthcare-2274523-supplementary.pdf]

Supplementary Material

Supplementary Figures and Tables

Table S1: Characteristics of the Study Participants

Table S2: Performance of Machine Learning Models to Predict UDM in the AoU Research Program

Figure S1. Classification error rate of RF model with respect to the number of trees

Figure S2. Learning curve of XGBoost model

Figure S3. Receiver Operating Characteristic (ROC) Curves of the ML Models for Female Population

Figure S4. Receiver Operating Characteristic (ROC) Curves of the ML Models for Male Population

Figure S5. Feature Importance for the Prediction of UDM among Females in AoU Research program 2023

Figure S6. Feature Importance for the Prediction of UDM among Males in AoU Research program 2023

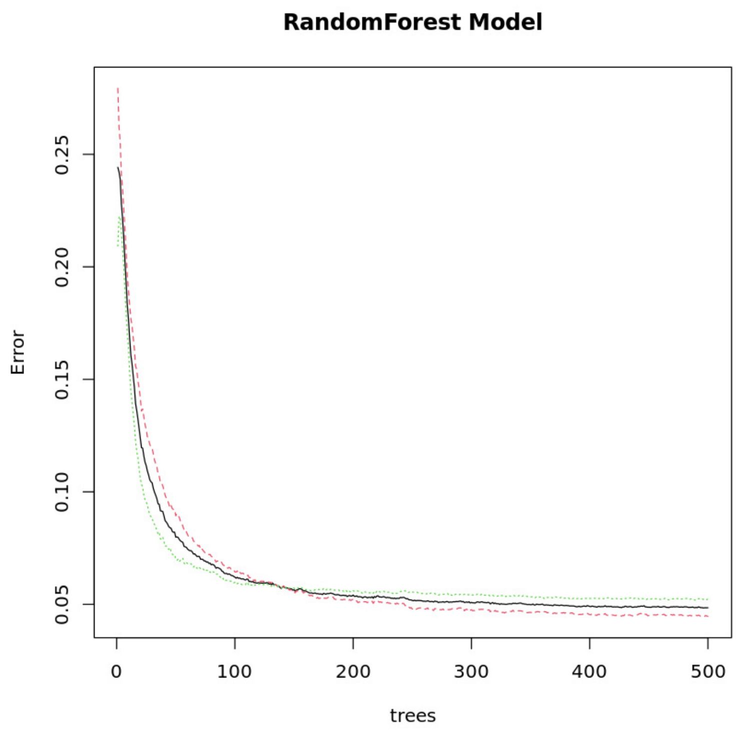

Figure S1. Classification error rate of RF model with respect to the number of trees

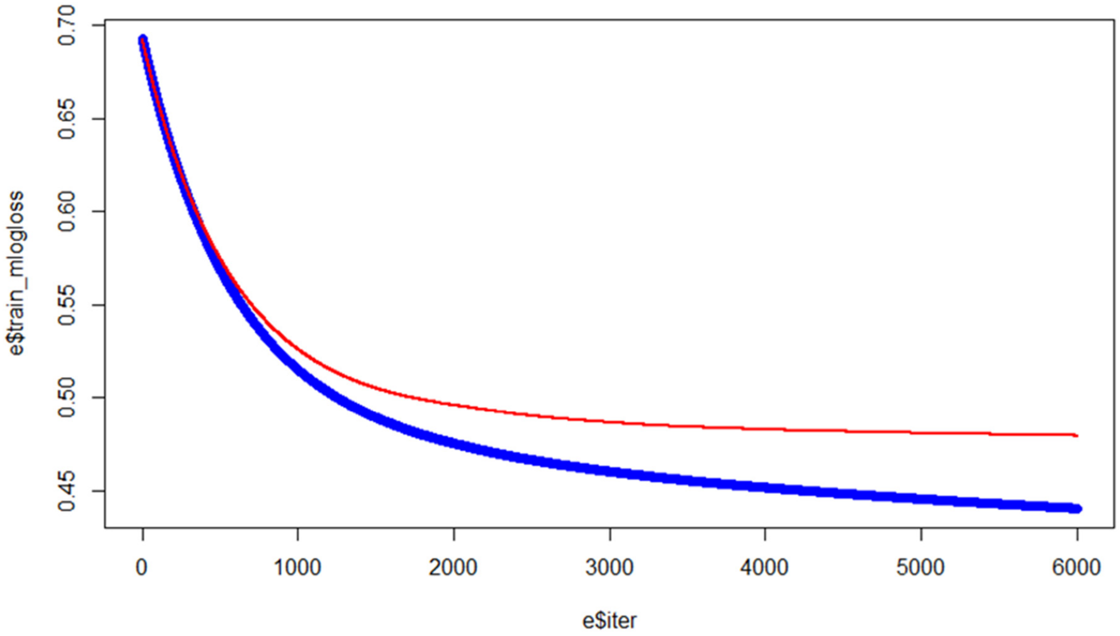

Figure S2. Learning curve of XGBoost model

The plot shows learning curves for the train (blue line) and test (redline) dataset where the x-axis is the number of iterations of the algorithm and the y-axis is the mlogloss of the model. Each line shows the mlogloss per iteration for a given dataset.

Table S1: Characteristics of the Study Participants

All continuous variables were described using mean values.

**Sociodemographic Characteristics**	Total: 33,826
**Gender**	
Male	10,744(31.76%)
Female	14,691(43.43%)
Unspecified	8,391(26.41%)
**Race**	
White	14873(43.96%)
Blacks	9000(26.6%)
Asian	632(1.87%)
Middle Eastern or North African	153(0.45%)
Others	9168 (27.1%)
Ethnicity	
Not Hispanic or Latino	24, 606(72.74%)
Hispanic or Latino	7627(22.55%)
Other	1593 (4.7%)
**Biomarkers**	
Continuous variables (mean)	
Age (mean/SD)	60(13.38)
Annual median income ($)	62,054.87(12927.96)
Deprivation index	0.33(0.1)

Chloride (mEq/L)	101.14(12.4)
Bicarbonate (mEq/L)	26.8(16.9)
Alanine transaminase (U/L)	29.63(17.1)
Albumin (g/L)	37.45(7)
alkaline phosphatase (IU/L)	87.6(26.7)
Anion gap (mmol/L)	10.73(3.2)
Aspartate aminotransferase (U/L)	26.62 (12.9)
Basophils (cells/µL)	5(1.9)
Bilirubin (mg/dL)	0.6(0.7)
Height (cm)	167.81(10.78)
Weight (kg)	94.54(24.8)
Calcium (mg/dL)	10.2(0.44)
Carbon dioxide (mEq/L)	25.3(2.37)
Total Cholesterol (mg/dL)	172.6(32.15)
HDL (mg/dL)	46.67(46.68)
LDL (mg/dL)	93.83(13)
Creatinine (mg/dL)	1.1(0.55)
DBP (mmHg)	76.57(8.63)
Eosinophil (cells/ µL)	23.68(58.3)
Erythrocytes (cells/ µL)	4.32(0.58)

Heart rate	78.91(10.42)
Leukocyte (cells/L)	8.26(2.43)
Lymphocytes (cells/ µL)	412.59(12.7)
MCH (picograms per cell)	29.157(2.13)
MCHC (g/dL)	32.98(1.12)
MCV (femtoliter)	88.11(5.38)
Monocytes (%)	6.9(1.47)
Neutrophils (%)	58(13.2)
Platelets (cells/ µL)	248.97(68.1)
Potassium (mmol/L)	7.1(0.53)
Respiratory rate	17.32(1.48)
Sodium (mEq/L)	138.27(2.36)
SBP (mmHg)	131(13.83)
Triglyceride (mg/dL)	156.2(68.82)
Urea nitrogen (mg/dL)	17.15(6.9)
**Clinical Characteristics**	
Controlled DM	26,281(80%)
Vomiting (yes)	7741
Myocardial infarction (yes)	3759
ACE Inhibitor/ARB (yes)	21470

Aspirin (yes)	20,771
Beta Blockers (yes)	17,134
Steroids (yes)	14,580
Acetaminophen (yes)	27,582
Statin (yes)	18,914
Opioids (yes)	20289
Nicotine (yes)	3,966
Arthritis (yes)	19,224
Polyuria (yes)	3,062
Paraesthesia (yes)	4,001

MCH: mean corpuscular hemoglobin, MCV: Mean corpuscular volume, MCHC: corpuscular hemoglobin concentration, DBP: Diastolic Blood pressure, SBP: Systolic Blood Pressure, LDL; Low Density Lipoprotein, HDL: High Density Lipoprotein

Table S2: Performance of Machine Learning Models to Predict UDM in the AoU Research Program

**Model Performance Based on Gender**				
**Both genders Combined**				
**Model**	**AROC**	**Recall**	**Precision**	**F1**
RF	0.77	0.95	0.81	0.77
XGBoost	0.75	0.80	0.85	0.82
LR	0.70	0.63	0.87	0.73
WEM	0.75	0.89	0.83	0.88
**Females**				
**Model**	**AROC**	**Recall**	**Precision**	**F1**
RF	0.78	0.96	0.81	0.86
XGBoost	0.74	0.88	0.83	0.85
LR	0.70	0.65	0.87	0.74
WEM	0.77	0.89	0.83	0.86

Model Performance Based on Gender				
**Both genders Combined**				
**Model**	**AROC**	**Recall**	**Precision**	**F1**
**Males**				
**Model**	**AROC**	**Recall**	**Precision**	**F1**
RF	0.75	0.97	0.80	0.88
XGBoost	0.69	0.88	0.82	0.85
LR	0.68	0.62	0.85	0.72
WEM	0.73	0.89	0.82	0.85

AoU: All of Us

LR: Logistic Regression

WEM: Weighted Ensemble Model

RF: RandomForest

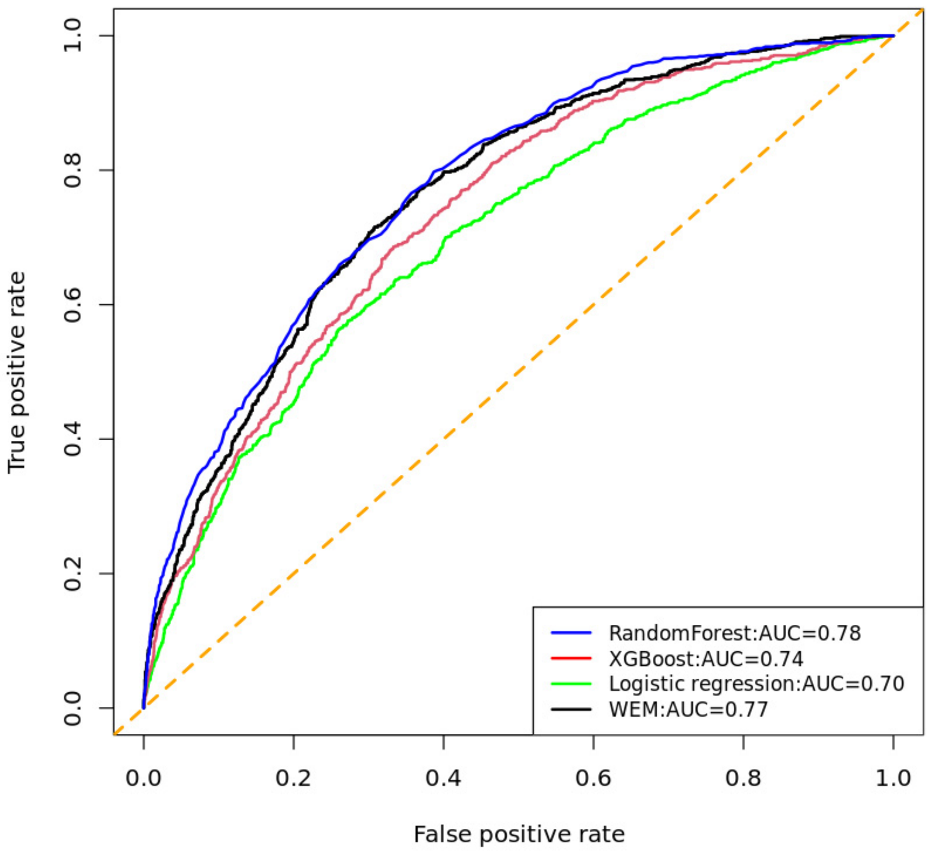

Figure S3. Receiver Operating Characteristic (ROC) Curves of the ML Models for Female Population

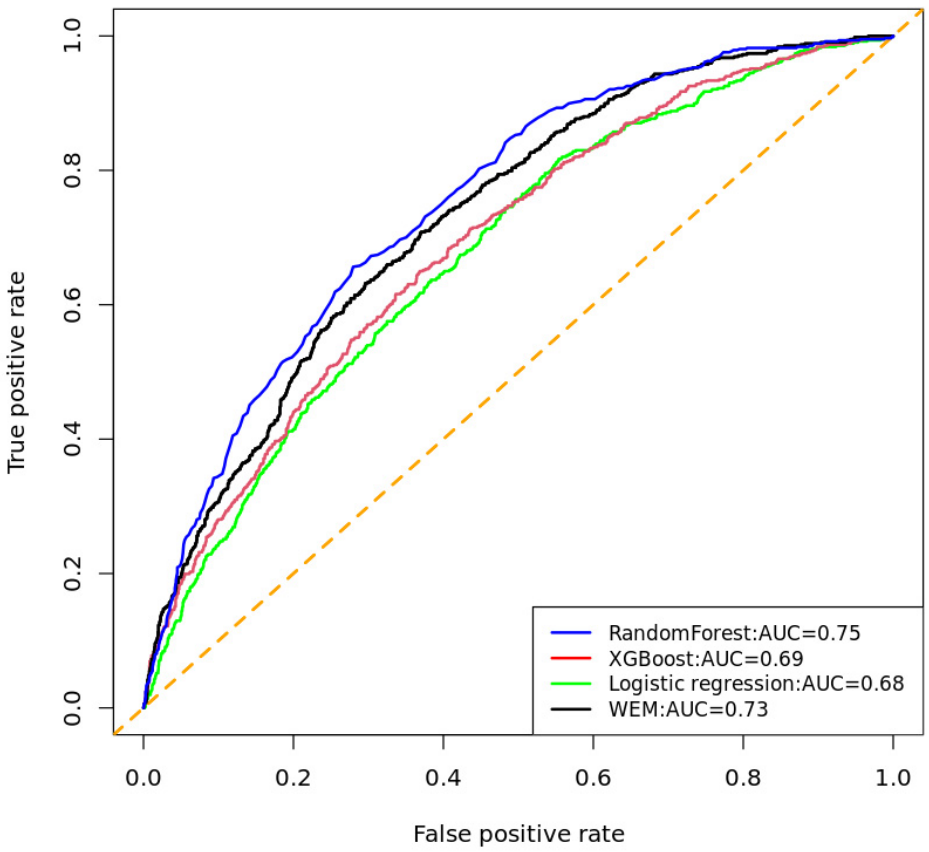

Figure S4. Receiver Operating Characteristic (ROC) Curves of the ML Models for Male Population

AUC: Area under the curve.

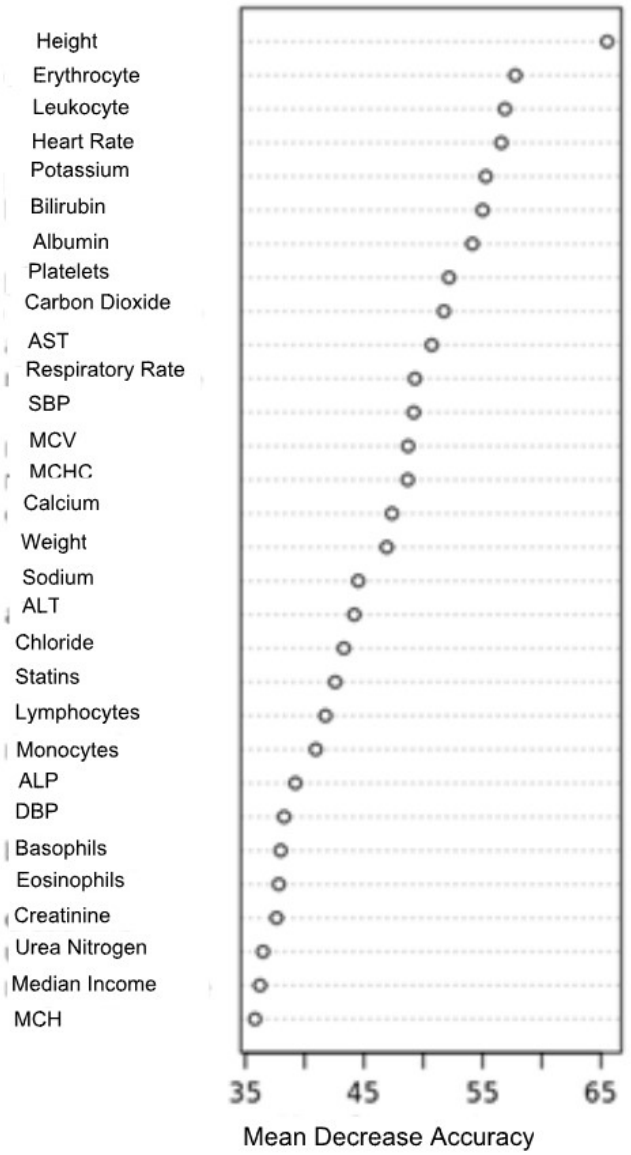
Height
Erythrocyte
Leukocyte
Heart Rate
Potassium
Bilirubin
Albumin
Platelets
Carbon Dioxide
AST
Respiratory Rate
SBP
MCV
MCHC
Calcium
Weight
Sodium
ALT
Chloride
Statins
Lymphocytes
Monocytes
ALP
DBP
Basophils
Eosinophils
Creatinine
Urea Nitrogen
Median Income
MCH
35
45
55
65
Mean Decrease Accuracy

Figure S5. Feature Importance for the Prediction of UDM among Females in AoU Research program 2023

AST: Aspartate aminotransferase, ALT: Alanine transaminase, ALT: alkaline phosphatase, DBP: Diastolic blood pressure, MCH: mean corpuscular hemoglobin MCHC: Mean corpuscular hemoglobin concentration, MCV: mean corpuscular volume, SBP: Systolic blood pressure.

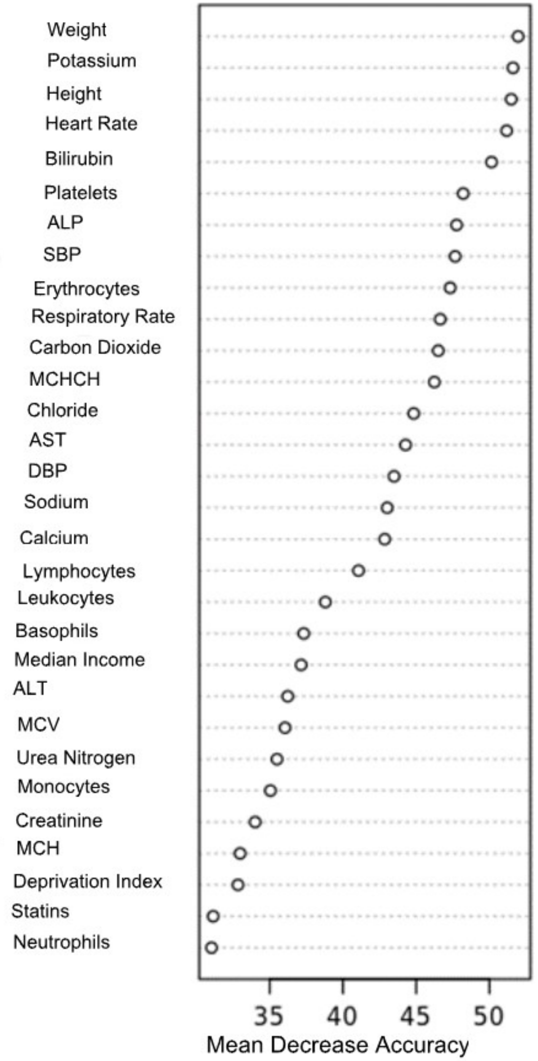

Figure S6. Feature Importance for the Prediction of UDM among Males in AoU Research program 2023

AST: Aspartate aminotransferase, ALT: Alanine transaminase, ALT: alkaline phosphatase, DBP: Diastolic blood pressure, MCH: mean corpuscular hemoglobin, MCHC: Mean corpuscular hemoglobin concentration, MCV: mean corpuscular volume, SBP: Systolic blood pressure.
